# Development of an Evidence-Based mHealth Weight Management Program Using a Formative Research Process

**DOI:** 10.2196/mhealth.2850

**Published:** 2014-07-11

**Authors:** Wilma Waterlander, Robyn Whittaker, Hayden McRobbie, Enid Dorey, Kylie Ball, Ralph Maddison, Katie Myers Smith, David Crawford, Yannan Jiang, Yulong Gu, Jo Michie, Cliona Ni Mhurchu

**Affiliations:** ^1^National Institute for Health InnovationSchool of Population HealthUniversity of AucklandAucklandNew Zealand; ^2^Wolfson Institute of Preventive MedicineQueen Mary University of LondonLondonUnited Kingdom; ^3^School of Exercise and Nutrition SciencesDeakin UniversityMelbourneAustralia

**Keywords:** weight loss, intervention, Internet, mobile phone, focus groups

## Abstract

**Background:**

There is a critical need for weight management programs that are effective, cost efficient, accessible, and acceptable to adults from diverse ethnic and socioeconomic backgrounds. mHealth (delivered via mobile phone and Internet) weight management programs have potential to address this need. To maximize the success and cost-effectiveness of such an mHealth approach it is vital to develop program content based on effective behavior change techniques, proven weight management programs, and closely aligned with participants’ needs.

**Objective:**

This study aims to develop an evidence-based mHealth weight management program (Horizon) using formative research and a structured content development process.

**Methods:**

The Horizon mHealth weight management program involved the modification of the group-based UK Weight Action Program (WAP) for delivery via short message service (SMS) and the Internet. We used an iterative development process with mixed methods entailing two phases: (1) expert input on evidence of effective programs and behavior change theory; and (2) target population input via focus group (n=20 participants), one-on-one phone interviews (n=5), and a quantitative online survey (n=120).

**Results:**

Expert review determined that core components of a successful program should include: (1) self-monitoring of behavior; (2) prompting intention formation; (3) promoting specific goal setting; (4) providing feedback on performance; and (5) promoting review of behavioral goals. Subsequent target group input confirmed that participants liked the concept of an mHealth weight management program and expressed preferences for the program to be personalized, with immediate (prompt) and informative text messages, practical and localized physical activity and dietary information, culturally appropriate language and messages, offer social support (group activities or blogs) and weight tracking functions. Most target users expressed a preference for at least one text message per day. We present the prototype mHealth weight management program (Horizon) that aligns with those inputs.

**Conclusions:**

The Horizon prototype described in this paper could be used as a basis for other mHealth weight management programs. The next priority will be to further develop the program and conduct a full randomized controlled trial of effectiveness.

## Introduction

There is a critical need for weight management programs that are effective, cost efficient, accessible, and acceptable to adults from diverse ethnic and socioeconomic backgrounds. Global rates of overweight and obesity have increased sharply in the last decades, and over 750 million individuals worldwide are estimated to be overweight and more than 300 million people are obese [1]. The global burden of disease attributable to excess body weight in adults continues to rise. Ischaemic heart disease and stroke collectively killed 12.9 million people in 2010 which relates to one in four deaths worldwide, compared with one in five in 1990. Moreover, compared to 1990, in 2010 twice as many deaths globally were due to diabetes [2]. The direct health care costs are estimated to be about 2-7% of annual health care budgets [3], but the true costs related to obesity are thought to be significantly higher [4]. Obesity and overweight show large and persistent social gradients and are more prevalent in disadvantaged socioeconomic groups in many Organisation for Economic Co-operation and Development (OECD) countries [5]. In New Zealand, the highest obesity rates (BMI>30 kg/m^2^) are reported among Pacific Islanders (64%), Māori (42%), and adults living in the most deprived areas (35-40%), compared with the population average of 25-26% [6].

Weight loss has been shown to improve many obesity-related illnesses and to reduce all-cause mortality [7]. However, the menu of evidence-based weight loss interventions currently available is limited. Pharmacotherapy has modest beneficial effects, but these are often lost once the medication is stopped [8]. Surgical interventions are more successful, but are expensive, unsuitable for large-scale use, and restricted to the morbidly obese [9]. Diet and/or physical activity interventions have variable effects [10], and interventions in primary care have reported mixed results [11]. More intensive behavioral interventions generate small but sustainable weight loss, which can engender clinically worthwhile long-term health benefits [12]. However, such interventions are generally limited to research contexts, are costly, and not widely available. Similar limitations apply to commercial weight loss programs such as Weight Watchers and Jenny Craig. While there is some evidence showing that these programs are effective, they are generally very intense and costly. Also, there is uncertainty whether the observed weight loss is clinically relevant and whether it would be sustained long-term [13]. A study by Cobiac et al, on the cost-effectiveness of Weight Watchers and the Lighten up to a Healthy Lifestyle Program revealed that such behavioral counseling interventions are not very cost-effective, and the potential benefits for population health are small [14].

International weight management guidelines (eg, New Zealand [15], United States [16], and Australia [17]) for overweight and obese adults recommend programs using “three key interventions in combination: changes to food/diet, increased physical activity, and behavioral strategies”. The Weight Action Programme (WAP) is a weight management program developed in the United Kingdom (UK) consistent with this multi-component, behavioral approach [18]. It entails a comprehensive package of cognitive, behavioral, and educational interventions including dietary advice, self-monitoring, exercise targets, and cue management, and is delivered in a group-based format over eight weeks. WAP was evaluated in two short-term pilots involving 162 overweight adults (mean BMI 35 kg/m^2^) from multiethnic areas of high deprivation in London. Average weight loss was 4.5 kg at 3-month follow-up [18]. Significant increases were also seen in physical activity levels, and knowledge of healthy eating.

While face-to-face approaches such as WAP or Weight Watchers are effective [19,20], they are relatively expensive to implement, difficult to scale up, and do not suit those who work or live some distance from venues. mHealth behavior change programs (delivered via mobile phones and Internet) offer a practical and potentially cost-effective solution to these barriers. There is broad population penetration of mobile and wireless technologies [21]. Globally, there were 6.8 billion mobile phone subscriptions in 2013, with mobile devices now exceeding traditional computers in unit sales [22]. In 2009, 89% of households in New Zealand had access to a mobile phone and in 2012, 80% had access to the Internet. There are no significant differences in Internet access across the country, gender, or level of education, and there are few differences in age below 65 years (>90% internet access in the age groups 15-34; around 90% in the age group 35-54; just below 80% in the age group 55-64; and around 60% in the age group 65-74) [23]. Previous research in New Zealand demonstrated strong support for an mHealth weight management intervention; with 75% of Māori and 65% of non-Māori saying they would use a mobile phone-based weight management intervention [24]. In line with this trend, a growing number of commercial programs including Weight Watchers and Jenny Craig deliver online/mobile tools as an alternative or extra support to their traditional programs. However, these mobile components are mostly an integrative part of the larger (traditional) programs and therefore still very expensive and the potential benefits for population health are small [14]. Also, it is unclear how effective these mobile components would be on their own (eg, separate from the traditional program components) and, as outlined in more detail below, there is a general lack of comprehensive randomized controlled trials on the effectiveness of these weight loss apps.

The aim of this study was to develop an evidence-based mHealth weight management program appropriate for ethnically and socioeconomically diverse target groups (Horizon), using a structured content development process (involving the target audience in the development stages) and formative research [25].

## Methods

### Development of the Horizon mHealth Weight Management Program

**Figure 1:**
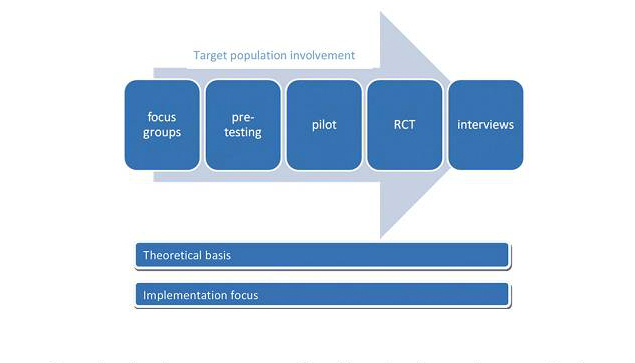
mHealth framework [25].

The development of the Horizon mHealth weight management program followed the steps of the mHealth framework (Figure 1) [25]. It involved the modification of the UK Weight Action Programme (WAP) for delivery via short message service (SMS) and the Internet. In this paper we report an iterative development process with mixed methods entailing the first two phases of the framework: (1) conceptualization using expert input on evidence of effective programs and behavior change theory; (2) formative research with target population input using focus groups and an online survey. Incorporating the findings from these phases, we present the prototype mHealth weight management program (Horizon).

### Expert Input

The project team included members from New Zealand (CNM, RW, RM), the United Kingdom (KMS, HMR) and Australia (KB, DC) with extensive expertise in nutrition, physical activity, mHealth, and behavior change. Conceptualization included review of existing WAP modules and key theoretical models of behavior change, including the Theory of Planned Behaviour [26] and the self-regulatory process action planning and coping planning [27]. The review also considered the outcomes of a meta-regression analyzing 122 behavior change interventions and classifying these interventions according to component and theoretical-based techniques [28]. This phase produced agreement on core components and functionality of the Horizon program, including mode of delivery.

mHealth programs can be delivered via various channels including texting, a website, and Smartphone apps. The expert panel decided that SMS text messaging and an interactive website would be the most appropriate mode of delivery for the Horizon program. There is already some evidence showing that SMS text messaging and Internet-delivered interventions have positive effects on some health-related behaviors, including physical activity [29,30] and weight-related behaviors [31]. Two recent systematic reviews support the use of texting [32] and Internet [30] interventions for achieving behavior change. Also, while the use of health-related smartphone apps has been rapidly increasing over the last several years (ie, in the Uited States, 674,000 free apps categorized in the “Healthcare and Fitness” category were downloaded via the Apple App Store in September 2013 [33]), there are some important limitations of these apps. A recent review conducted by our team (unpublished data) assessed the concordance of weight loss apps with the New Zealand clinical guidelines for weight management [15] and revealed that of the 20 most popular weight loss apps just 22% met the New Zealand guidelines criteria. Few apps included important components such as a comprehensive approach (including food, activity and behavior) or emphasized “permanent weight management change”. None of the apps reported any testing or evaluation with consumers, and no published research on the apps could be found. Other studies found similar results by showing that weight loss apps typically included only a minority of the behavioral strategies found in evidence-based weight loss interventions [34]. Other disadvantages of weight loss apps include the requirement for ownership of a smartphone or tablet and the fact that they rely on consumer initiative to use them. SMS text messaging on the other hand, is reactive, more direct and can be delivered in a timely manner as initiated by the program not the consumer. Also, SMS text messaging is available on every type of mobile phone and has great penetration into low socioeconomic populations.

In addition to the SMS text messaging module, it was decided that Horizon should include an interactive website and a hard copy toolkit. The website was important for additional effective program components including self-monitoring of progress and peer support. Likewise, since text messages only allow 120 characters, it was decided to develop a hard copy toolkit which participants could refer to for more detailed information. These three modules (eg, texting, website, and toolkit) were the basis for target population input.

### Target Population Input

Target population input was obtained by focus group and one-on-one phone interviews which were used to develop the initial content of the program modules (ie, text messages, toolkit and webpages) followed by an online survey to obtain in-depth feedback on the proposed content.

#### Focus Group and Phone Interviews

Three focus groups were conducted in Auckland, New Zealand, supplemented with five one-on-one phone interviews. Purposive sampling methods were used to recruit participants via advertisements in local newspapers and posters in community venues. A local mailbox drop was also conducted. Phone interview participants (n=5) were recruited via the assistance of the Department of Pacific Health at the University of Auckland. Inclusion criteria were that people had to be aged 18 years or older, overweight (BMI≥25), own a mobile phone, and want to lose weight. Participants needed to be able to provide informed consent and to converse in English. All participants received a gift voucher for their participation.

The focus group interviews were carried out according to standardized procedures. All focus groups were audiorecorded and conducted by a psychologist experienced in facilitating focus groups and qualitative research methods. Prior to the interviews, basic demographic information including ethnicity, gender, and age was recorded. During the interviews, an effort was made to involve all participants and they were encouraged to express their opinions. The focus groups followed a semi-structured format (allowing for unscripted ideas to surface and for a reflexive discussion to take place) and lasted approximately 60 minutes each. Their purpose was to gain in-depth information on participants’ past experiences of weight management, and their perceptions around an mHealth weight management program. Topics that were explored included: mobile phone usage, positive and negative experiences of previous weight management attempts, perceived acceptability of the proposed mHealth program (barriers and benefits), preferred style of messages, frequency of messages and feedback on specific components of the program (eg, interest in being able to text a question). After the three focus groups, a preliminary analysis was carried out and results were fed back to the study investigators. At this point it was decided that further information was needed around family dynamics and more Pacific Islander input into the program development was required. Questions on family shopping/cooking were added. Five phone interviews were subsequently carried out with Pacific Islanders by the same interviewer. These were also audiotaped and the same semi-structured interview technique was used.

Following transcription, all data (focus groups and phone interviews) were analyzed using a general inductive thematic approach to identify common themes and meanings. NVivo9 software was used to manage the transcripts and facilitate the analysis. Data were read and re-read as they were collected. Each focus group and phone interview was coded separately. Once all data were collected, categories and subcategories were organized and refined. Subsequently, all categories were grouped into themes and discussed with members of the research team.

#### Online Survey

The results of the focus group interviews were used to inform the development of the initial content of the Horizon program modules (eg, text messages, toolkit, and website). Subsequently, a quantitative online survey was conducted to obtain in-depth feedback on drafts of the module content and delivery format. Particular emphasis was placed on aspects such as preferred text message frequency, integration of social support, and response to different message styles and content. Participants were recruited via similar methods used for the focus group interviews supplemented with email networks and the aim was to include n=150 participants. The survey was undertaken using LIME software (open source survey application). Data were analyzed and summarized using descriptive statistics and findings were reported back to the expert group to refine development of the prototype program.

## Results

### Expert Input

Review of existing WAP modules and key theoretical models of behavior change determined that the core techniques for the program should include: (1) self-monitoring of behavior; (2) prompting intention formation; (3) promoting specific goal setting; (4) providing feedback on performance; and (5) promoting review of behavioral goals [28]. These techniques were integrated in each of the modules of the program; the text messages, the website and the hard copy toolkit. Moreover, it was agreed that the small steps approach for weight management should be an integral part of the program. Recent findings suggest that it is more motivating for people to set small short-term goals that they can successfully achieve (eg, eat one piece of fruit per day), instead of failing large long-term goals (lose 10 kg of weight in the next month). Also, small steps are more likely to evolve into a sustainable healthy lifestyle (eg, creating a habit) [35].

### Target Population Input

#### Focus Group and Phone Interviews

There were 20 participants (18 female; 2 male) in three focus groups (5 to 8 participants per group), and 5 (2 female; 3 male) participated in the phone interviews. Most (n=16) were New Zealand European, two were Māori (indigenous), and all phone interviewees were of Pacific Island ethnicity. The majority of focus group participants were aged between 35 and 64 years (n=17).

**Table 1:** Focus group themes.

Theme #	Focus group themes
1	Weight management is a roller coaster
2	More than just energy-in/energy-out: psychological factors
3	A lifestyle change
4	Goal setting: set us up for success
5	Support us on all levels
6	Cultural considerations
7	Perceived benefits and barriers of mHealth

During focus groups, seven themes emerged (Table 1). When participants were asked to describe positive and negative experiences they had when trying to manage their weight (Theme 1), they were unable to clearly differentiate between the two. For most, attempts to manage weight were described as always starting off positive and then ending negatively. Many had tried several different weight management programs (eg, Weight Watchers, Jenny Craig, etc). These programs were generally described as having unrealistic goals and being not sustainable. Some felt these programs were boring, repetitive, expensive, and involved eating processed tasteless food. For some, having to go to meetings and get weighed in front of others was described as embarrassing and degrading as they felt they were being judged. One participant described a workplace competition to lose weight that has been working well for her as she liked the nonconfrontational positive support she received from her colleagues. Group support was a feature that was predominantly found to be valuable, at least in the first instance while it was still novel.

I can’t afford it and I found Weight Watchers was really successful for about a year and then it became too boring and you just go to those meetings and you listen to the same old stories, the same old people, same people talk – it’s just nothing’s new, nothing, it’s not that motivating. And the other thing I’ve tried is the Green Prescription, which is the reduced cost one that you can get through your GP but the only times you can do it are during work hours, that’s the only time that the gym is available and things like that so it’s completely out of limits, off limits to people who work.

The majority of participants felt they knew and had repeatedly heard educational information around weight management (Theme 2). They discussed that despite knowing what they “need to do”, they still struggled to make changes and expressed an awareness of there being more to weight management than exercising and eating healthy. Words that came up throughout discussions of weight management included guilt, shame, embarrassment, emotional eating, feeling judged, secret eating, and bingeing.

I would imagine that quite a few of us here, have um, we’re all overweight and we all know we are, and we all know how to eat healthy and everything like that. I’m sure somewhere inside there’s something that’s just not letting us mentally.

There was a strong desire among all participants for something to be developed that could guide them as to how they make sustainable lifestyle changes (Theme 3). They wanted a long-term cost-effective solution for not only themselves, but also for their families. Also, they would like practical tips on exercise and useful recipes to cook at home.

I think it’s best to have menus and food or dishes that one are the run of the mill kind of foods that we would generally eat, whereas to make it so that the whole family can eat those so that you’re not having separate things. I think when you have separate, first of all it creates a culture within the family that you’re different and that you’ve got a problem and they don’t. And it also sets up um a kind of understanding that in fact they can eat all the bad things and you can’t, and so later on you’re probably causing weight problems for your kids and people in your family.

Participants expressed feeling fed up due to not succeeding at weight management programs (Theme 4). The “all-or-nothing” strategies they had tried in the past left them feeling demoralized when they either failed to lose weight, hit a plateau, or regained the weight they had lost. They expressed a desire for a program that was realistic and would allow them to slip up once in a while.

It’s not a 100% stick to a diet, it’s really got to be 75% and you can fall off the wagon. If you’re going to tell me I’ve got to be 100% then I think we’re all kidding ourselves.

When discussing goal setting, almost all participants expressed a preference for lifestyle-based goals, with their level of effort reflected in recognition of achievement of their goal. Participants were predominantly against the sole use of weight-related goals, but were open to having a small and realistic weight goal (for example, ½ kg per week) alongside lifestyle-based goals. The use of body mass index (BMI) as a way of prescribing behavior change and measuring success was perceived negatively, as often being daunting and unachievable, and not taking into consideration their individual situation and level of fitness.

Family support for weight management was not something that was desired, although participants could see the benefit of a family attempt (Theme 5). A few participants said that in the past, their family members had sabotaged weight loss attempts and that they preferred support from professionals and from others going through similar experiences through an online forum, group meetings, or a buddy system where they can text each other. Additionally, the majority of participants did not want to be involved in another program which would tell them educational weight management information they already knew. One described this as patronizing. Another participant stated:

So I don’t want nutritional information. Other people might disagree. I want the little person on my shoulder telling me have a little bit more strength and motivation. As you say, motivation or inspiration or whatever it is.

In contrast, a few participants expressed that although they had heard many of the educational health messages before, it would be beneficial to be reminded of them. There was also a desire to have conflicting messages clarified by a credible professional. They liked the concept of being able to text someone with questions regarding physical activity or healthy eating. However, there was a strong preference for an immediate reply at the moment they needed support. Professional support was also wanted in the form of someone tracking their progress on meeting goals.

None of the five Pacific Island participants that were interviewed had engaged in any of the organized weight management programs (Theme 6). Adapting lifestyle changes was seen as something that needed to be done at the family level. In terms of physical activities, one participant suggested that it would be best to have a variety of options for people to choose from. He made the suggestion that traditional dance classes may appeal to some people as they are fun and undertaken in a “safe” environment. It was thought by one participant that an mHealth program might be very suitable to Pacific Islanders who may not feel comfortable in a public weight loss support group.

Yeah I think as a um Pacific Islander they’re not really into, most Pacific Islanders are pretty laid back and they’re not confrontational, they’re not comfortable sitting face to face with people and having discussions. And this type of message will go a lot easier, well it will be more acceptable to them than sitting and talking with somebody face to face because they’ll feel shy and they’ll say yes, yes, yes and all the time they don’t really want to do this type of thing.

Two of the phone interview participants expressed an interest in testimonial stories of people who have been through similar ups and downs and are now successfully making lifestyle changes.

Mobile phones are the technology that most people were comfortable with and the majority of participants were very positive and excited about the potential of an mHealth program (Theme 7). Nonetheless, a few concerns about such an approach were raised, including technological limitations (eg, areas with poor network coverage), lack of tailoring, and potential reduced accountability to a phone.

#### Online Survey

A total of 171 people accessed the online survey of which 120 (70.17%) consented and completed the questionnaire (Table 2). Of these, 18 reported having normal weight and two reported not owning a mobile phone. These were excluded from further analysis. Of the 118 mobile phone owners, 41% owned a smartphone.

Of all the participants, 13.7% (16/117) said that they would like to receive program text messages less frequently than daily, the remainder expressed preferences for once a day (44/117, 37.6%) or more often (57/117, 48.7%). Almost all participants (115/119, 96.6%) said that they would reply to text messages if asked.

**Table 2:** Participant characteristics online survey (n=120).

Characteristics			n (%)
**Sex**			
	Male		16 (13.3)
	Female		104 (86.7)
**Age**			
	18-24		10 (8.3)
	25-34		27 (22.5)
	35-44		36 (30.0)
	45-54		28 (23.3)
	55-64		16 (13.3)
	65+		3 (2.5)
**Ethnicity**			
	New Zealand European		34 (28.3)
	Māori		53 (44.2)
	Pacific Island		22 (18.3)
	Asian		5 (4.2)
	Other		6 (5.0)
**Weight (self reported)**			
	Normal		18 (15.0)
	Overweight		102 (85.0)

Ten sample text messages were presented to the participants with only one message receiving more than 12.5% (14/112) negative feedback. For the message content, participants reported that these should have a personal touch, give tips straightaway (eg, not just a link to another source), use trustworthy language (eg, no texting language/abbreviations) and offer peer support. With regard to the toolkit (hard copy folder with additional information), 13 potential information items were proposed and for all items a large majority (>86%) of participants said that they would like to see information on these items included (Table 3). The two most popular items were “Snacks: cutting down, healthy options” and “Healthy, quick and easy recipes”. In addition, participants requested information on reading nutrition labels, local options for physical activity, tools to track progress, and information on the energy content of foods.

**Table 3:** Responses to proposed items in the toolkit (descending order).

Item	Should be included (yes) (n/n) %
Snacks: cutting down, healthy options	(116/116) 100.0
Healthy, quick, and easy recipes	(107/110) 97.3
How to cut down portion sizes and energy intake	(108/112) 96.4
Temptation and how to deal with it	(112/117) 95.7
How to cook and eat healthy when you don’t have time	(108/113) 95.6
Exercise, physical activity, TV watching	(106/113) 93.8
Fat, sugar, carbs and protein: understanding the basics	(104/113) 92.0
My personal plan and goals for a healthy life	(106/116) 91.4
Breakfast: fuel yourself for the day	(100/114) 87.7
Drinks: more water and less sugar	(97/112) 86.6
Energy (calorie) content of common foods	(99/114) 86.8
5 pieces a day: eating more fruits and vegetables	(94/109) 86.2
Myth busting	(97/113) 85.8

### Horizon Prototype Development

The outcomes of the expert and consumer studies were used to develop the Horizon prototype weight management program. The Horizon prototype comprises three main modules: (1) text messages; (2) toolkit; (3) website. These three modules all link together (eg, the text messages refer to information in the toolkit) and are designed in line with the five core components (eg, self-monitoring of behavior; prompting intention formation; promoting specific goal setting; providing feedback on performance; and promoting review of behavioral goals [28]).

**Figure 2:**
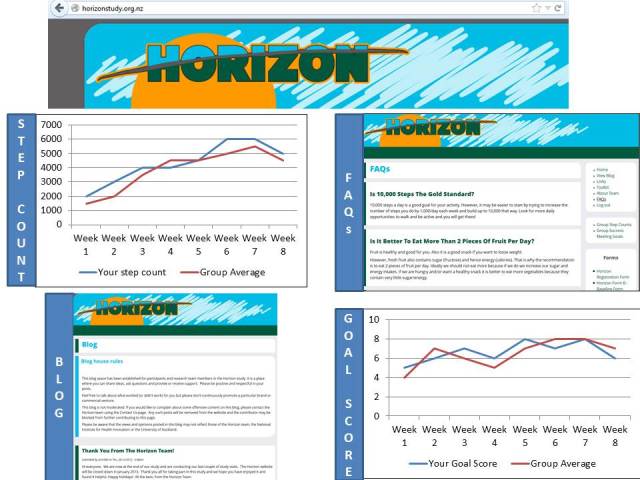
Horizon website.

A library of 130 different text messages which focus on motivation, goal setting, getting social support, monitoring and use of other study components (eg, toolkit and website) was developed with the goal of sending participants an average of two text messages per day (Table 4). The text messages are designed to encourage participants to set new well-specified goals each week (such as “I will bring fruit to work and eat this every day with morning tea”) and are tailored according to whether the participant is the main household shopper/cook and/or whether the participant has children. At the end of each week, participants receive a monitoring text message asking them to review how well they accomplished their goal and to text a number from 1 (not successful at all) to 10 (very successful). This information will be displayed graphically on the Horizon website, showing the participants’ individual score compared with the average score of the entire study population. In a similar way, participants will be asked to text in their step count for graphical display on the website (Figure 2). In addition to these monitoring and motivational components, participants will be provided with the opportunity to send their questions using text messages to the study team which will be answered within 24 hours.

**Table 4:** Text messages in the Horizon prototype for the first week.

Text category/type^a^	Example
Motivational/small steps	*Small steps add up to important changes. This is not a quick fix but developing new habits to last a lifetime*
Goal setting/reference to toolkit/ Motivational/monitoring behavior	*It is important to set small goals each week. The toolkit has suggestions. Pick one goal to get started on this week, write it down and go for it!*
Pedometer/monitoring behavior	*Ralph^b^ here, wearing your pedometer and recording your daily step count in your toolkit is a great way to track your progress, start today!*
Motivational/monitoring behavior	*Hi (name), we are here to support you along the way. Each week we will ask about your step count and goals, and you can track your progress online.*
Monitoring behavior	*Cliona^b^ here, writing down what you eat can help you understand your eating patterns. Try it for the next 7 days to see where you can make changes*
Reference to toolkit/reference to website/monitoring behavior	*Hi (name), this is Cliona your nutrition coach. You may find the food diary and the weight tracker in the toolkit to be useful tools.*
Motivational/goal setting/small steps	*Have you reduced the sugar in your tea and coffee? It can taste funny at first but you will soon get used to it. Try it now. Cliona*
Getting support	*Hi (name), where can you get support? Making changes is easier with the help of others. See if your friends or family will join in too.*
Pedometer/monitoring behavior/reminder	*Hi this is Ralph your activity coach. Have you been wearing your pedometer? I will text you in 3 days for your daily step count.*
Getting support/reference to the website	*If you are finding it hard to get people to support your efforts, ask others on Horizon to help via the horizon study weblog*
Motivational/goals setting	*Hi (name), remind yourself daily why you are doing this by writing your reasons somewhere you can see them everyday.*
Motivational/getting support/ reference to the toolkit/reference to the website	*Did you read about Jamie in the toolkit/online. He removed all snacks and sweets from his house to help him in the early days.*
Monitoring success	*Hi. Overall, how well do you think you did with your goal this week? Reply with a number from 1 (not well at all) to 10 (brilliantly).*
Motivational/reference to the toolkit	*Cliona again. If you are starting to get bored with your healthy meals, the toolkit has recipe websites to inspire new ideas.*

^a^ The messages were slightly tailored; we developed specific texts according to: (1) whether the participant was the main household grocery shopper/cook; (2) whether participants had children or not.

^b^ Horizon includes a physical activity coach (Ralph) and a nutrition coach (Cliona) who were incorporated in the program to give it a more personal feel.

The toolkit serves as a source of more detailed information and contains eleven sections, including: goals and tips, personal plan (writing down weekly goals), monitoring resources (eg, food diary, step count diaries, weight records), recipes, and stories of successful weight loss. The toolkit focuses on ideas for short-term goals and practical suggestions on how to reach these goals. The website serves as a further source of information, and focuses on providing a blogging space to enable participants to share their stories and experiences with each other and the researchers, and provides a graphical display of their self-monitoring of daily step counts and success in reaching the weekly behavioral goals (Figure 2).

Following this study, the Horizon program will be further refined and subsequently tested in a full randomized controlled trial of the effectiveness of the final Horizon program.

## Discussion

### Principal Findings

The aim of this research was to develop an mHealth weight management program based on behavior change techniques, proven effective weight management programs, and closely aligned with participants’ needs and preferences [25]. The resulting Horizon prototype program involved modification of the UK Weight Action Programme (WAP) for delivery via SMS text messaging and the Internet. Expert review of the evidence of effective programs determined that core components of a successful program should be: (1) self-monitoring of behavior; (2) prompting intention formation; (3) promoting specific goal setting; (4) providing feedback on performance; and (5) promoting review of behavioral goals.

We started our development by applying the theory and philosophy behind WAP, the specified necessary components, and our experience in behavior change support via technology, to delivery of a program via mobile phones and the internet. Subsequent target group input confirmed that participants liked the concept of an mHealth weight management program and would like the program to have a personal touch, immediate and informative text messages, practical and localized physical activity and dietary information, culturally appropriate language, social support (group activities or blogs), and weight tracking functions. These individual constructs (eg, self-efficacy; goal setting) and environmental characteristics (eg, localized information; social support) have previously been identified as crucial in developing effective physical activity [36] or dietary interventions, and confirm the complexity of developing successful behavior change programs [37].

Our study revealed that the majority of participants were very positive and excited about the potential of an mHealth program. The sample text messages (presented to the online panel) received very positive feedback and the majority of participants expressed preferences for at least one text message per day. Moreover, 97% of participants indicated they would reply to a text message if asked to do so. These results are promising and show that mHealth could potentially provide a practical and cost-effective alternative to face-to-face approaches (eg, WAP or Weight Watchers [19,33]) that are relatively expensive to run, difficult to scale up, and do not suit those who work or live some distance from venues.

### Strengths and Limitations

A limitation of our study was that the ethnic mix was not as diverse as originally intended; thus we conducted an additional five phone interviews with Pacific people. However, the online survey was successful in recruiting an ethnically diverse sample. Results showed that Horizon would appeal to different ethnic groups, including Pacific Islanders who may not feel comfortable in a public weight loss support group.

The strength of our structured iterative development process [25] is that the program builds on effective programs and behavior change techniques, ensures that the program is engaging and useful to the target audience (by including target audience input at early development stages and by evaluation of prototypes), and then follows up with gold standard research methods to determine the effectiveness of the intervention [25]. In publishing the findings of this development process and formative research, we envisage that this information will be useful to other developers and researchers, and that our process is replicable in significantly different target populations and adaptable across multiple platforms and technologies. However, an important limitation of this development process is the time required. In this case, the formative research and prototype development took six months. A randomized controlled trial of effectiveness would add at least another 2 years to the total timeframe. Technology continues to evolve at a rapid pace and the way people use technology continues to change over time [25]. By the time evidence on the effectiveness of Horizon is obtained, the technology and mode of delivery might be outdated. While existing smartphone apps are likely to have been developed more rapidly, our research-based user-informed development process aims to deliver a more proven and acceptable intervention. A second potential issue comes from building on insights from behavior change theories developed for other less immediate and on-going, less proactive and less dynamic media. Whether these theories hold with innovative technological advances in mHealth is yet to be determined [38]. The success of using a new technology is likely to depend on its ease of use, engagement, and level of appeal to different consumer groups. Therefore, it seems relevant to consider other models, such as research and development processes used by successful consumer technology product companies.

The mHealth components used in the Horizon prototype (eg, text messages and an interactive website) are technologies that most people feel comfortable with and have widespread access to. However, as we progress our development, we will consider also modification for a smartphone app to allow participants to opt for the technology that suits them. The Horizon prototype can as such be viewed as a basis for other mHealth weight management programs and can be updated in accordance with the newest technological innovations as required.

### Conclusions

mHealth weight management programs have the potential to assist in weight loss; however, well-designed high-quality pilot and subsequent scaled up RCTs evaluating the long-term benefit and cost-effectiveness of interventions employing mobile technology are needed. This paper describes an iterative development process to create a feasible and acceptable mHealth weight management program. The presented prototype (Horizon) can be used as a basis for future mHealth weight management programs and we envisage that this information is useful to other developers and researchers.

## Abbreviations

BMI: body mass index
OECD: Organisation for Economic Co-operation and Development
SMS: short message service
WAP: Weight Action Program
